# Immunoglobulins and Transcription Factors in Otitis Media

**DOI:** 10.3390/ijms22063201

**Published:** 2021-03-21

**Authors:** Su Young Jung, Dokyoung Kim, Dong Choon Park, Eun Hye Lee, Yong-Sung Choi, Jeewon Ryu, Sang Hoon Kim, Seung Geun Yeo

**Affiliations:** 1 Department of Otorhinolaryngology-Head and Neck Surgery, Myongji Hospital, Hanyang University College of Medicine, Goyang 10475, Korea; monkiwh35@hanmail.net; 2 Department of Anatomy and Neurobiology, College of Medicine, Kyung Hee University, Seoul 02447, Korea; dkim@khu.ac.kr; 3 Department of Gynecologic Oncology, St. Vincent’s Hospital, The Catholic University of Korea, Suwon 16247, Korea; park.dongchoon@gmail.com; 4 Department of Pediatrics, College of Medicine, Kyung Hee University, 23 Kyungheedae-ro, Dongdaemun-gu, Seoul 02447, Korea; leeeh80@gmail.com (E.H.L.); feelhope@gmail.com (Y.-S.C.); 5 Department of Otorhinolaryngology-Head and Neck Surgery, College of Medicine, Kyung Hee University, Seoul 02447, Korea; jeewon@bu.edu (J.R.); hoon0700@naver.com (S.H.K.)

**Keywords:** otitis media, acute otitis media, otitis media with effusion, chronic otitis media, immunoglobulin, antibody

## Abstract

The causes of otitis media (OM) involve bacterial and viral infection, anatomo-physiological abnormalities of the Eustachian canal and nasopharynx, allergic rhinitis, group childcare centers, second-hand smoking, obesity, immaturity and defects of the immune system, formula feeding, sex, race, and age. OM is accompanied by complex and diverse interactions among bacteria, viruses, inflammatory cells, immune cells, and epithelial cells. The present study summarizes the antibodies that contribute to immune reactions in all types of otitis media, including acute otitis media, otitis media with effusion, and chronic otitis media with or without cholesteatoma, as well as the transcription factors that induce the production of these antibodies. The types and distribution of B cells; the functions of B cells, especially in otorhinolaryngology; antibody formation in patients with otitis media; and antibodies and related transcription factors are described. B cells have important functions in host defenses, including antigen recognition, antigen presentation, antibody production, and immunomodulation. The phenotypes of B cells in the ear, nose, and throat, especially in patients with otitis media, were shown to be CD5^low^, CD23^high^, CD43^low^, B220^high^, sIgM^low^, sIgD^high^, Mac-1^low^, CD80(B7.1)^low^, CD86(B7.2)^low^, and Syndecam-1^low^. Of the five major classes of immunoglobulins produced by B cells, three (IgG, IgA, and IgM) are mainly involved in otitis media. Serum concentrations of IgG, IgA, and IgM are lower in patients with OM with effusion (OME) than in subjects without otitis media. Moreover, IgG, IgA, and IgM concentrations in the middle ear cavity are increased during immune responses in patients with otitis media. B cell leukemia/lymphoma-6 (Bcl-6) and paired box gene 5 (Pax-5) suppress antibody production, whereas B lymphocyte inducer of maturation program 1 (Blimp-1) and X-box binding protein 1 (XBP-1) promote antibody production during immune responses in patients with otitis media.

## 1. Introduction

**Figure 1:**
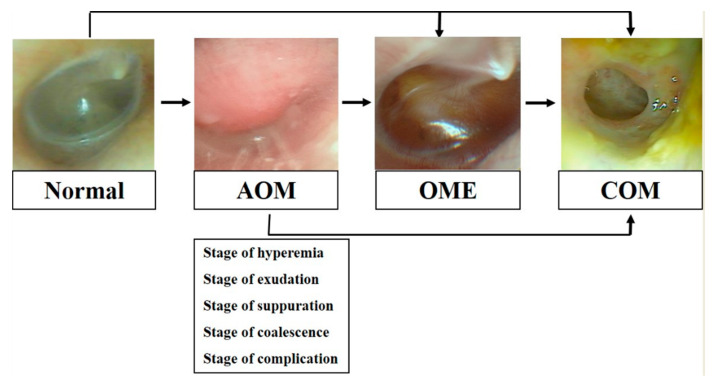
The progress of otitis media (OM).

Otitis media (OM) refers to all inflammatory phenomena that take place in the middle ear [1]. OM is classified as acute if its duration is less than 3 weeks, subacute when it lasts more than 3 weeks but less than 3 months, and chronic if it lasts more than 3 months. The disease can also be classified based on the presence or absence of perforation in the tympanic membrane and the form of otorrhea/ear discharge. OM with effusion (OME) is defined as an absence of perforation coupled with the accumulation of inflammatory fluid in the middle ear, whereas chronic suppurative OM is defined as the presence of both perforation and suppurative discharge. Chronic OM (COM) can be categorized as COM with or without cholesteatoma depending on the presence of cholesteatoma. In most patients, acute OM heals without complications, but some patients experience a relapse of inflammation or OME. COM may also develop if the inflammation is not treated sufficiently (Figure 1) [1,2].

The causes of acute OM are very diverse and are associated with complex interactions of various factors, including infection with viruses or bacteria, malfunction of the Eustachian tube, allergy, physiological/pathological/immunological factors within the middle ear, and environmental and genetic factors [2]. The factors responsible for the development of chronic inflammation in patients with acute inflammation of the middle ear and mastoid have not yet been clarified.

Acute OM (AOM) and OME can cause structural changes in the tympanic membrane, with histologic alterations observed in the fibrous layers of the lamina propria. These alterations affect the elasticity of the tympanic membrane, creating conditions that can result in retraction or perforation of the tympanic membrane. COM that develops in adulthood may also be the result of AOM. The mechanism underlying the conversion of AOM to COM is not yet clear, but risk factors for the development of AOM and OME may also be risk factors for chronicity. Acute inflammation of the middle ear causes pathological transformation and hyperplasia of the middle ear mucosa. This hyperplasia, as well as the influx of various inflammatory cells into the mucous membrane, is mostly reversible. After the stimulation associated with otitis media disappears, the mucous membrane recovers to its normal shape through de-differentiation. However, the repeated occurrence and chronicity of pathologic conditions, such as hyperplasia of the middle ear mucosa, middle ear effusion due to hyperproliferation reactions, atelectasis, tympanosclerosis, and middle ear cholesteatoma, can cause irreversible structural changes in the middle ear cavity. Although AOM is usually cured without sequelae, some patients may experience recurrent inflammation, resulting in recurrent otitis media or persistent OME, leading to the development of COM [3,4].

There have been significant advances in the treatment of OM due to the development of antibiotics. Although antibiotics have reduced critical complications of OM, they have not reduced the frequency of occurrence, with some patients experiencing serious complications. About 10% of patients with OM develop COM. These patients may develop complications, including conductive/sensorineural hearing loss, tympanic membrane perforation, retraction pocket/atelectasis, tympanosclerosis, ossicular discontinuity/fixation, mastoiditis/petrositis, labyrinthitis, facial nerve paralysis, cholesterol granuloma, infectious eczematoid dermatitis, postauricular abscess, Bezold’s abscess, zygomatic abscess, lateral sinus thrombophlebitis, meningitis, extradural abscess, subdural abscess, brain abscess, or otitic hydrocephalus [5,6].

The development of OM involves the interactions of various bacteria; viruses; epithelial, inflammatory, and immune cells; and effusions. Moreover, these factors may respond to each other in a complex manner. Important inflammatory mediators in OM are involved not only in the invasion of immune cells, such as neutrophils, monocytes, and lymphocytes, but also in interaction with local cells such as keratinocytes and mast cells [7]. The present study therefore sought to identify the antibodies related to immune reactions to external antigens in the middle ear and the transcription factors that induce the production of antibodies.

Literature databases, including SCOPUS, PubMed, the Cochrane Library, and EMBASE, were searched for studies published in English. Studies were included if they (1) were prospective and retrospective investigational studies; (2) included patients diagnosed with acute OM, OME, COM without cholesteatoma, or COM with cholesteatoma, while excluding patients with complications of OM; and 3) included human patients only. Keywords searched included OM, acute OM, OME, COM without cholesteatoma, COM with cholesteatoma, immunoglobulin, and antibody.

## 2. Types and Functions of B cells

### 2.1. Types and Distribution of B cells

Humans possess five types of immunoglobulin (Ig), IgG, IgA, IgM, IgD, and IgE, although concentrations of IgD and IgE are very low. The biological characteristics of these antibodies include neutralization of toxins, immobilization of bacteria, condensation of bacteria and antigen particles, precipitation of soluble antigens enabling their phagocytosis by macrophages, binding to bacteria facilitating cytolysis by serum complement, and destruction of bacteria by phagocytes and cytotoxic T lymphocytes [8].

Both acute and chronic inflammation of the tympanic cavity result in the production of antibodies by B lymphocytes, cells involved in antigen recognition, antigen presentation, antibody formation, and immunomodulation and that possess receptors for IgM and IgD, and the surface markers cluster of differentiation 19 (CD19), CD20, and CD21. Antibodies are present, but are distributed unevenly, in body fluids, including blood and cerebrospinal fluid (CSF), the spleen, the thymus, and peripheral lymphoid tissues and are involved in humoral immunity. The ratio of B lymphocytes to T lymphocytes differs among tissues and organs. B lymphocytes are rarely found in the thymus, with the ratio of T lymphocytes to B lymphocytes in this organ being 8:1. This ratio is 1:1 in the spleen and 1:3 in the CSF. Flow cytometry has shown that the ratio of B lymphocytes to T lymphocytes in mouse cervical lymph nodes is 1:2.8 ± 0.76 [7].

B lymphocytes can be classified as B-1 and B-2 cells. B-1 cells express the glycoprotein CD5 on the pan-T cell surface, distinguishing them from B-2 cells. In addition to CD5, B-1 cells express surface Ig (sIg) M^high^, sIgD^low^, B220^low^, CD23^low^, and CD43^high^ on their surfaces, whereas B-2 cells express (sIg)M^low^, sIgD^high^, B220^high^, CD23^high^, and CD43^low^ but do not express CD5. Most B lymphocytes in the spleen are B-2 cells, whereas most B lymphocytes in the abdominal cavity and thoracic cavity are B-1 cells [9,10,11].

Morphologically, B-1 cells are larger but less dense than B-2 cells. More than 90% of fetal lymphocytes are B-1 cells, but this percentage decreases with age, with B-1 cells constituting 25~35% of B cells and 0~6% of total lymphocytes in adults [5,6].

B-1 cells constitute 50~80% of the B lymphocytes obtained by perfusion of the abdominal cavity of newborn mice and 20% of the B lymphocytes in the spleen [12]. In comparison, B-1 cells constitute 5% of the B lymphocytes in adult mice, with almost none of these cells present in the lymph nodes [12]. Following CSF transplantation, B-1 cells are the first to proliferate and increase in immunodeficiency states during the immune reconstruction stage, suggesting that B-1 cells play a role as a primitive immune system. The abundance of B-1 cells in newborns is regarded as a primary immune mechanism of early natural immunity, as these cells are produced during early stages of immune system development and are especially increased when newborns are infected [9,13].

### 2.2. Functions of B cells

B cells have several important biological functions, including antigen recognition, antigen presentation, antibody production, and immune regulation. B cells are categorized into CD5-positive B-1 cells and CD5-negative B-2 cells. B-1 cells constitutively secrete IgM, which not only contributes to natural immunity but also reacts with autoantigens and is significantly increased in patients with chronic lymphatic leukemia and several autoimmune diseases. Stimulation of B cells by T cells, by cytokines produced by T cells, or by T-independent antigens results in the proliferation, differentiation, and apoptosis of B cells, which ultimately differentiate into effector cells, either plasma cells or memory cells. Stimulators used most often in basic science research include CD40L, lipopolysaccharide (LPS), and interleukin-4 (IL-4). As T-dependent antigens, CD40L and IL-4 play important roles in homing and localization of B lymphocytes in lymphoid organs through the proliferation and differentiation of B lymphocytes, as well as adherence, the transition of immunoglobulin types, and induction of cytoskeleton activation. LPS, a T-independent antigen, can stimulate the activation, proliferation, and differentiation of B cells without involvement of other cells or cytokines [14,15].

The B-lymphocyte proliferation and differentiation pathways include several maturation phases prior to their final differentiation into plasma cells that produce antibodies. Plasma cells that differentiate from B cells initially produce immunoglobulins M and D, followed by DNA rearrangement and class switching to produce immunoglobulins G, A, or E [16]. B-1 cells and B-2 cells differ in their constitutive production of immunoglobulins. For example, B-2 cells in the spleen do not constitutively produce immunoglobulins, whereas B-1 cells in the abdominal cavity produce IgM in the absence of stimulation. B-1 cells not only secrete IgM constitutively; some of these cells are also precursors of plasma cells that secrete IgA, which is involved in immunity of mucous membranes including that of the intestines. Immunoglobulins derived from B-1 cells have fewer mutations and short nontemplated N-insertions, thus limiting the number of these cells, inasmuch as the immunoglobulins produced by these cells are generally closer to the germline state than the immunoglobulins produced by B-2 cells. Immunoglobulins produced by B-1 cells can distinguish among the factors composing bacterial cell walls, suggesting that B-1 cells are implicated in specific germlines or produce natural antibodies that provide serological defense against microorganisms prior to immune responses induced by microorganisms. Natural immunoglobulins can limit the spread of pathogens and can play a major role in the survival of infected hosts [17,18]. These types of natural immunoglobulins, however, are not produced only by B-1 cells in the abdominal cavity and do not remain confined to the abdominal cavity. Rather, they can migrate to the spleen as their Mac-1 phenotypes are diminished and produce natural IgM at this site. Moreover, splenic B-2 cells stimulated by antigens possess the phenotype of B-1 cells. These cells can move to the abdominal cavity, suggesting that the spleen plays an important role in maintaining balances of B-1a and IgM production [19,20].

B cell proliferation usually begins with primary B cells, which normally remain in the G0 phase of the cell cycle. These cells move into the S phase when the cycle is stimulated by a metabolic change caused by the cross-reaction of an antigen with immunoglobulin receptors on the surface of B cells. Although B-1 cells were found to be generally unresponsive to anti-Ig stimulation, B-2 cells showed active progression through the cell cycle in response to anti-Ig [21]. This difference was attributed to the activation of sufficient phospholipase C of B-2 cells compared with the non-activation of phospholipase C of B-1 cells and problems with regulation of signal transduction mediated by CD5-associated Src homology region 2 domain-containing phosphatase 1 (SHP-1). Nevertheless, phorbol ester stimulation of thymidine incorporation was found to peak after 24~30 h in B-1 cells, whereas phorbol ester did not stimulate the proliferation of B-2 cells. Rather, thymidine incorporation in B-2 cells peaked 54–60 h after the addition of calcium ionophore to phorbol ester, rather than after 24~30 h. This weak or negligible response of B-2 cells to phorbol ester was likely due to the absence of cyclin D2 production and the inability of cyclins D2 and D3 to form a complex with cyclin dependent kinase 4/6. Further, although phorbol ester is capable of forming a cyclin D3-cdk complex in B-2 cells, it was unable to stimulate the phosphorylation of the retinoblastoma tumor suppressor gene (pRb) [21,22,23]. Given that phospholipase C and CD5-associated SHP-1 are activated in splenic B-2 cells, but these cells do not produce cyclin D2, the proliferative responses of B-2 and B-1 cells in the abdominal cavity differ following the stimulation of surface receptors on B cells.

## 3. B cells in Otorhinolaryngologic Fields

Various studies have established that B cells in the ear, nose, and throat are involved in the production of antibodies, including the secretion of IgA. Another study reported, however, that these cells were more involved in the production of IgG, a disparity thought to be due to immune system differences in selected tissues and the functions of these tissues. The distributions of B-1 cells and B-2 cells or the type of stimulation may influence the type of immunoglobulin produced and the amount secreted. IgM and IgG are normally produced in nasal polyps, with IgA occasionally being secreted. Most of the B cells in the middle ear mucosa secrete IgA, with a small number of cells producing IgM [24,25]. In contrast, more of the CD5+ and CD5- B lymphocytes present in the adenoids produce more IgG than IgA or IgM [26].

A study of murine expression factors involved in the differentiation and proliferation of B cells found that the phenotype of B cells in cervical lymph nodes was CD5^low^, CD23^high^, CD43^low^, B220^high^, sIgM^low^, sIgD^high^, Mac-1^low^, CD80 (B7-1)^low^, CD86 (B7-2)^low^, and Syndecam-1^low^, as shown by flow cytometry-double immunofluorescent labeling, enzyme-linked immunosorbent assay (ELISA), and [^3^H]thymidine incorporation assay. Ig was not constitutively produced during cell differentiation. IgM was secreted in response to LPS stimulation, with IgA and IgG observed on Day 5. Active cell proliferation was observed through the S (synthetic) phase on Day 2 of CD40 and anti-CD8 stimulation [27,28].

A flow cytometry study investigating the distribution and frequency CD5+ B cells, γδ T cells, and CD56+ NK cells, which are involved in natural immunity, during idiopathic adenoid and tonsillar hypertrophy, found that more cells were stained with anti-CD5 monoclonal antibodies than with antibodies to γδ T cell receptors and CD56. CD5-positive cells were usually located in interfollicular and subepithelial sections, with some of these cells also observed in follicles, the follicular mantle, and the epithelium. Most CD5-positive cells in the epithelium and subepithelium were located near the stratum basale of the epithelium and at the junction between the epithelium and subepithelium. The numbers of CD5-positive cells differed significantly in sections of the tonsils, with the number being higher in the follicular mantle than in follicular areas (*p* < 0.01). CD5-positive cells were also present in the epithelium and subepithelial sections of the normal pharyngeal mucosa of posterior pillars, but there were fewer cells at these locations than in tonsillar tissues. The percentages of CD19-positive cells in children that were also CD5-positive were similar in palatine tonsils (19.8 ± 8.7%), adenoids (24.8 ± 14.1%), and blood (21.1 ± 9.6%). In adults, the percentages of CD19-positive cells that were also CD5-positive were also similar in palatine tonsils and blood (15.6 ± 7.2% vs. 19.3 ± 10.6%, *p* = 0.89). In addition, the mean fraction of CD5-positive cells in blood was similar in adults (19.3 ± 10.6%) and children (21.1 ± 9.6%). Taken together, these findings indicate that CD5+ B cells are abundant in tonsillar tissue [29].

Since the adenoids are located in the upper wall of the nasopharynx, these organs are always in contact with allergy-inducing antigens via air breathed through the nose. Some of these antigens on mucous membranes are transferred to the nasopharynx by ciliary movement and to the lower part of the pharynx by swallowing behavior. The mucosa moves from the nasal and paranasal cavities directly to the adenoids covered by the ciliated epithelium. This trait allows allergy-inducing antigens to be in contact with immune cells, one of the main components of adenoid tissue [30]. In the adenoids, B lymphocytes are mainly distributed in the germinal center and columnar layer, with most B lymphocytes in the germinal center being activated B lymphocytes and most in the columnar layer being stabilized or memory B lymphocytes. Immunohistochemical assays of IgE expression in adenoid samples obtained from patients with and without allergic rhinitis who underwent surgery for adenoid hypertrophy found that IgE was expressed near the germinal centers and submucosal regions in both groups, and that staining intensity and extent in four selected areas did not differ significantly [31].

## 4. Antibody Formation in Otitis Media

Five classes of Igs are present in blood: IgG, IgA, IgM, IgD, and IgE, with IgG constituting 75% of Igs in blood. IgG, the only immunoglobulin that can pass through the placenta, can be categorized into four subclasses [32]. The normal proportions of these IgG subtypes are IgG1 60~70%, IgG2 20%, IgG3 10%, and IgG4 5% [33]. IgG is produced starting at birth, with concentrations of IgG1 and IgG4 reaching adult levels at age 7–8 years; and concentrations of IgG3 and IgG2 reaching adult levels at ages 10 and 12 years, respectively [34]. IgG1 is the main subtype that forms antibodies against viral protein antigens, and IgG2 is the main subtype against *Streptococcus pneumoniae* (Spn), *Haemophilus influenzae* (Hi), and polysaccharide antigens [33].

IgA constitutes about 15% of Igs and is usually located in mucous membranes of the nasopharynx, providing primary defense against local inflammation. The quantity of IgA present in mucous membranes is greater than that of all other classes of Ig. IgA can be divided into two subclasses, IgA1 and IgA2, and can form secretory IgA (sIgA) dimers, which are present in tears, saliva, sweat, and colostrum, as well as in mucosal secretions of the genitourinary tract, gastrointestinal tract, prostate, and respiratory epithelium. The secretory element of IgA protects these proteins from degradation by protein hydrolases in the gastrointestinal tract environment and from microorganisms that proliferate in body secretions [6]. sIgA can suppress the inflammatory effects of other Igs [35]. sIgA primarily binds to ligands in pathogens and inhibits their binding to epithelial cell receptors [36]. IgA reduction results in lower production of antibodies against pneumococcal polysaccharide, which influences recurrent infection. Moreover, IgA deficiency may occur temporarily in children. IgA concentrations increase relatively slowly in children, with deficiency diagnosed after age 2~3 years. IgA deficiency is defined as 14~159 mg/dl at age <5 years, and 33~236 mg/dl at age 6~10 years.

IgM constitutes about 10% of serum immunoglobulins and is involved in most humoral immune reactions, especially to bacteremia [26]. IgM antibodies are the first to be secreted by B cells following antigen stimulation, including during early stages of infection, and reappear, but at lower concentrations, upon re-exposure to antigen [32]. Unlike IgG, IgM cannot pass through the placenta. IgM is useful in diagnosing infectious diseases, as the presence of IgM in a patient’s serum indicates a recent infection.

Igs are among the most important defenses against pathogen invasion and the resulting upper respiratory infection, such as OM. Expression of Ig is closely associated with disease activity, with many studies reporting differences in expression between serum and middle ear fluid (MEF) (Table 1).

Acute OM (AOM) is an acute inflammatory disease in the tympanic cavity caused primarily by bacterial or viral infection. Since immunoglobulins are important in defenses against bacterial infections, immunoglobulin expression patterns have been assessed in patients with AOM. A study comparing the levels of expression of IgG, IgM, and IgA in MEF and serum from 255 patients with AOM found that the levels of IgG and IgM were higher in serum, whereas the level of IgA was higher in MEF. These findings suggested that the MEF in these patients primarily represented a secretory response to inflammation rather than a transudate. In addition, infants older than 9 months of age who showed higher concentrations of IgA in MEF were generally culture-negative, but the exact mechanism remained unclear [37]. Similarly, studies have compared concentrations in serum and MEF of antibodies against pathogens such as Hi and Spn, which are considered primary causes of AOM and targets of vaccination. A study comparing the concentrations of pneumococcal antibody serotypes 1, 3, 6, 14, 18, 19, and 23 in MEF and serum of 61 children with AOM found that, during the acute phase of disease, IgG and IgM were predominant in serum, whereas IgG, IgM, and IgA were all detected in MEF. In addition, the concentrations of IgG, IgM, and IgA were increased during the convalescent phase of AOM. The detection of significant concentrations of IgA in MEF during the acute phase of AOM suggests that IgA is involved in the inflammatory process in the middle ear of children with AOM. Moreover, the finding that the three classes of immunoglobulin were increased in serum during the convalescent phase suggests that the systemic immune response is involved in the pathophysiology of AOM [38]. Similar results were observed in 40 children with AOM caused by Hi, with IgG being predominant in serum samples and high ratios of IgG, IgM, and IgA to Hi concentrations in serum and MEF. The concentrations of IgG and IgA in MEF were higher than the concentration of IgM, with the IgA antibody being more frequently observed in MEF of patients lacking the IgA antibody in serum. These findings suggested that young children aged <2 years with OM responded both systemically and locally to Hi by producing specific antibodies [39]. A similar trend was observed in patients with AOM induced by viral pathogens. Although IgG was predominant in serum, the concentration of IgA was more than 4-fold higher than that of IgG in MEF. Additionally, the virus-specific IgA concentration was found to be higher in patients vaccinated against viruses. Taken together, these reports all demonstrate the existence of local immune responses against inflammatory events in the tympanic cavity of patients with AOM. Moreover, a significant increase in IgA concentrations in vaccinated individuals suggests that vaccines are effective in children with AOM [40]. In another study, children with AOM caused by Hi and Spn were divided into groups with cleared and uncleared MEF, and the concentrations of IgG, IgM, and IgA in MEF were compared. The concentrations of the three classes of immunoglobulins were higher in the cleared MEF than in the uncleared MEF group, suggesting that the clearance of Spn or Hi from MEF was significantly associated with the presence and concentration of specific antibodies in MEF at the time of diagnosis [33].

Another study investigated whether colonization of causative pathogens affects the expression of Igs. That study, which compared serum antibody concentrations against *Moraxella catarrhalis* (Mcat) in 35 AOM patients and 149 healthy controls, found that specific IgG antibodies against Mcat were detected in all serum samples regardless of AOM occurrence. IgG antibodies against outer membrane proteins (OMP) were significantly higher during the convalescent phase of AOM. In addition, serum concentrations of IgG antibodies against oligopeptide permease (OppA), Moraxella surface protein (Msp)22NL, and hemagglutinin (Hag)5-9 were lower when Mcat colonized the nasopharynx, suggesting that high levels of antibody against these three proteins were correlated with reduced carriage [41].

Another study sought to identify the origins of Igs found in MEF by comparing the concentrations of IgG and IgA in nasal wash (NW) fluid, MEF, and serum of 137 patients with AOM. IgG concentrations were higher in MEF and serum than in NW, whereas IgA concentrations were highest in NW. The similar patterns of expression in serum and MEF suggested that Igs in MEF originate by diffusion from serum rather than by reflux through the Eustachian tubes from the nasopharynx, whereas sIgA in MEF likely derived from local immune responses in the MEF [42]. Another study assessed the serum IgG titer against Spn according to recurrence and response to treatment. In that study, involving 34 patients with AOM, 35 with recurrent AOM (rAOM), and 25 with AOM treatment failure (AOMTF), the serum anti-Spn IgG concentration was lowest in the rAOM group during the acute phase, suggesting that the lower immune response in this group could increase the likelihood of AOM recurrence. The serum anti-Spn IgG concentration was highest in the patients with non-recurrent AOM during the convalescent phase, suggesting that lower levels of production of anti-Spn IgG could increase the risks of recurrence and treatment failure [43]. Similar results were observed in assays of IgG against nontypeable Hi (NTHi). The concentration of serum anti-NTHi IgG was lowest in the rAOM group during the acute phase, whereas the concentration of anti-protein D (anti-PD) was significantly higher only in the non-recurrent AOM group during the convalescent phase. These findings suggested that anti-PD IgG could protect patients with AOM due to NTHi from AOM recurrence and treatment failure [44]. The serum concentrations of total IgG (IgG1, IgG2), IgM, and IgA were found to be significantly lower in children who were than those who were not prone to recurrent OM. Serum IgA, IgM, IgG, and IgG1 concentrations in each age group were similar in children with and without OM or were higher in those affected by OM due to antigen stimulation, whereas IgG2 concentrations were generally lower in children with OM [34]. Similarly, IgG and IgG1, but not IgG2, titers against pneumococcal polysaccharides were higher in 166 patients with rAOM than in 61 healthy controls, with trends being similar in serum and MEF [45]. Another study found that serum and MEF concentrations of anti-NTHi IgG were higher in patients with rAOM than in healthy controls [46], perhaps because recurrent infection in the rAOM group consistently stimulated the immune system to produce high concentrations of antibodies. In particular, IgG2, which was found at low levels in the rAOM group, is an important primary defense factor against Spn and Hi, as these antibodies opsonize capsular polysaccharides. Therefore, the low concentrations of IgG2 in the rAOM group support the limited ability to defend against pathogens leading to rAOM. However, when serum IgG2 concentrations and the frequency of respiratory tract infection were measured in adults who had shown low IgG2 as children, these adults showed normal levels of IgG2 and no increase in the frequency of respiratory tract infections [47]. Similarly, IgG2 levels are higher than IgG1 levels in healthy adults, whereas IgG1 levels are higher in children. In addition, IgG2 concentrations are lower in children with prone-OME (pOME) than in age-matched healthy controls [48]. These findings suggest that low IgG2 levels in childhood could be associated with an increased frequency of rAOM in children, but that IgG2 concentrations were normal in adults through growth and normal age-related increases. Thus, despite differences in childhood, these groups show similar patterns of defenses against respiratory tract infections as adults.

A study comparing serum and MEF levels of IgG, IgM, and IgA antibodies against Spn and Mcat in children with rAOM and chronic OME (cOME) found no between-group differences in the concentrations of these antibodies, whether in serum or MEF. Moreover, immunoglobulin concentrations were independent of bacterial species. However, only IgG concentrations in serum and MEF were strongly correlated in both the rAOM and cOME groups. These findings suggested that ET function or environmental factors play more important roles than immunoglobulins in the pathophysiology of rAOM and cOME. Thus, immunoglobulins may be less potent in patients with repeated infection and those who develop chronic OME. These discrepancies with previously described studies indicate the need to evaluate additional influences, such as ET function or environmental factors [49].

Studies have also investigated the effects of IgG, IgM, and IgA concentrations in MEF on chronicity and recurrence of OM. The concentration of IgA in MEF has been reported lower in patients with pOME than OME, suggesting that a lower IgA concentration affects the chronicity and recurrence of OME. Bacterial stimulation of immunity in the middle ear of patients with AOM has been found to increase the concentration of IgA. However, defects in secretory Ig production and disorders in local defense mechanisms may decrease IgA concentrations, leading to the recurrence and chronicity of OME [50]. Concentrations of Ig-immune complexes (ICs) in MEF were found to vary when patients with OME were classified as having acute, subacute, and chronic phases according to disease activity. The concentration of IgG-ICs was the highest in the acute and chronic phases, whereas the concentration of IgA-ICs was the highest in the subacute phase. These results suggested that immune complexes in the tympanic cavity may play an important role in the prolonged inflammatory process of OME by activating the complement following the chemotaxis of neutrophils [51].

A comparison of IgG, IgM, and IgA concentrations in serum of cOME patients and normal controls found that the concentrations of all three immunoglobulins were lower in the cOME group than in the control group. The inflammatory reactions in cOME are regarded as chronic rather than acute. Since Ig concentrations were low in the cOME group, OME was regarded as not improving in its initial stage; rather, it continues to persist in the form of cOME. In addition, analysis of Ig expression in the serum and MEF of cOME patients showed that Ig concentrations in MEF did not correlate with the species of bacteria or serum Ig concentrations, with serum Ig concentrations being higher in patients in whom bacteria were identified than in those in whom bacteria were not identified [49]. Differences in the patterns of immune responses in MEF and serum may be responsible for differences in responses in effusion fluid and serum samples obtained from individual patients. Thus, the high serum Ig concentration observed when bacteria were positively identified may be due to the effect of systemic immunity in patients with cOME. In contrast, because the Ig concentration in effusion fluid was not affected by bacterial identification, immune reactions in effusion fluid are less influenced by systemic immunity than immune reactions in serum. Alternatively, immune reactions in effusion fluid may be independent of systemic immune reactions. Other studies of serum concentrations of total IgG, IgG subclasses, total IgA, and IgA subclasses in OME patients aged >3 years showed that all of these concentrations did not differ between control and OME groups, and that each concentration did not differ from that in age-matched normal controls. Regardless of OME, both groups showed similar aged-matched normal antibody responses. Moreover, the contribution of systemic immune reactions to the pathophysiology of OME was likely relatively small in each age group [53].

Ig expression patterns were also compared in MEF and serum. MEF has been categorized as serous and mucoid types, with the sIgA concentration being higher in the mucoid type. Mucoid-type MEF may show a stronger immune response than the fluid type, with the viscosity of MEF increased due to the production of various inflammatory products, including mucin, lysozyme, and IL-8 [54]. A study of Ig expression patterns in MEF of 59 children with OME showed that the expression level was high, in the order IgG > IgM > sIgA > IgA, with the concentration of all sampled Igs being higher in MEF than in serum. IgG and IgM showed the most increased pattern in the acute phase, and sIgA was increased in the subacute or chronic phase. Additionally, serum and MEF IgG were lower in patients with recurrent OME than patients with non-recurrent OME. Taken together, these findings suggested that the continuation and recurrence of OME were due to reduced IgG in serum and MEF [55]. Similarly, the IgG concentration was higher than IgA and IgM in both serum and MEF, and IgA was higher than IgM in MEF. These findings suggested that OME induces local immune responses in the tympanic cavity through the activation of IgA in MEF [56].

Another study compared IgG and IgM concentrations in serum and MEF of patients with acute suppurative OM (aSOM), chronic suppurative OM (cSOM), and a control group. Serum IgG concentrations were higher in the cSOM than in the control group, but lower than in the aSOM group. Serum IgM concentrations were higher in both SOM groups than in the control group. These findings suggested that chronic and repetitive inflammatory responses could enhance the production of serum IgG in patients with cSOM, as well as somewhat increasing the production of serum IgM in patients with aSOM and cSOM, as these enhancements usually indicate recent infection. This hypothesis was supported by findings showing that IgM concentrations in MEF were higher in aSOM than in cSOM patients, whereas IgG concentrations were higher in MEF of patients with cSOM [57]. In addition, serum IgE concentrations were highest in cSOM patients, as well as being somewhat higher in aSOM patients than in controls. MEF IgE concentrations were also higher in cSOM patients, showing significant correlations with serum IgE concentrations. These findings suggested that IgE-related allergy appears to play a contributory role in cSOM and that elevated IgE in MEF is indicative of a likely mucosal response [58].

In summary, the patterns of increases and decreases in Igs concentrations as a function of the type of OM or disease activity are diverse. Moreover, they suggest that local immune responses in the middle ear may be independent of systemic immune responses in the tympanic cavity.

## 5. Antibodies and Related Transcription Factors in Otitis Media

Following antigen stimulation, B cells can differentiate into plasma cells in germinal centers, producing high-affinity antibodies and often surviving for several months in the bone marrow. Four transcription factors, B cell leukemia/lymphoma 6 (BCL6), B lymphocyte inducer of maturation program-1 (BLIMP-1), paired box gene 5 (PAX5), and X-box binding protein 1 (XBP1), have been associated with the production of Ig and play major roles in the process by which LPS stimulates B-2 cells to differentiate into plasma cells [59,60].

BCL6 and PAX5 suppress antibody production and differentiation into B cells and plasma cells in germinal centers, whereas BCL6 and XBP1 facilitate differentiation by suspending the cell cycle. The transcription repressor BCL6, which is involved in B cell differentiation and is highly expressed in germinal centers, represses the cyclin-dependent kinase inhibitors p27 and p21 to suppress the rapid cell differentiation. The main functions of BCL6 are its inhibition of BLIMP-1 and its suppression of the differentiation of B cells into plasma cells.

BLIMP-1 induces the expression of cdk inhibitor 18; the proapoptotic genes GADD45 and GADD153, which are required for differentiation of B cells into plasma cells; and J chain, XBP1, and HSP-70, which are involved in Ig secretion [61].

A study of transcription factors involved in antibody production in MEF obtained during surgery for OME found that the expression of BLIMP-1 and IgA was significantly lower in the OME-prone than in the non-prone OME group, suggesting that the reduction in antibody production in response to reduced BLIMP-1 expression would reduce immunity against pathogens and contribute to the recurrence and chronicity of OME [50].

PAX5 is required for differentiation of germinal center B cells into plasma cells and for inhibition of antibody production. PAX5 possesses dual activities, as it can activate or suppress gene transcription. PAX5 inhibition increases XBP1, which is needed for immunoglobulin and heavy chain secretion. PAX5 elevation suppresses the differentiation of B cells into plasma cells, reducing antibody production [62].

Although defects in XBP1 result in the production of normal T cells and B cells, the formation of normal germinal centers, and normal cytokine production, Ig production is severely impaired. Thus, XBP1 is crucial for the differentiation of B cells into plasma cells. XBP1 expression was significantly lower in patients prone than not prone to OME, as well as being associated with reduced antibody production and increased recurrence of OME [50].

The levels of expression of BLIMP-1 and XBP1 were significantly lower in patients who were prone than those who were not prone to otitis, whereas expression of BCL6 and PAX5 tended to be higher in the otitis-prone group. These results suggest that BCL6 and PAX5 expression suppresses antibody production, whereas BLIMP-1 and XBP1 expression promotes antibody production in the middle ear, and that impaired production of antibodies against invading pathogens in the tympanic cavity is more related to the recurrence and chronicity of OM [50].

## 6. Conclusions

OM is one of the most common diseases in infants and children, with significant social and economic costs. OM may lead to language development disorders, delayed language acquisition, aprosexia, and behavioral abnormalities. It is also a representative otologic disease, in that recurrent or chronic OM can induce otological symptoms, including hearing loss, otorrhea, tinnitus, and ear fullness at all ages. OM can result in various complications, including those regarding the temporal bone, and requires surgical treatment. Many studies have attempted to identify factors that induce OM, as well as inflammatory responses, inflammatory mediators, and innate and acquired immune responses, enabling the progressive elucidation of the pathogenesis and pathophysiology of OM.

The occurrence of otitis media is accompanied by the production of B cell-related antibodies during acute and chronic inflammation of the middle ear cavity. Of the five classes of immunoglobulins produced by B cells, three (IgG, IgA, and IgM) are produced in patients with otitis media. Phenotypically, B cells in otitis media are B-2 cells that express sIgM^low^, sIgD^high^, B220^high^, CD23^high^, and CD43^low^. Immunoglobulins are among the important defense mechanisms in upper respiratory infections such as otitis media, and the expression of appropriate immunoglobulins to protect against pathogens invading the body is closely related to disease activity. Immunoglobulin expression patterns, including differences between expression in serum and middle ear fluid, have been found to differ in patients with acute otitis media, otitis media with effusion, and chronic otitis media with or without cholesteatoma. In particular, lack of production of antibodies in serum and middle ear fluid due to otitis media can result in hearing loss, aggravation of symptoms, chronicity of otitis media, and increases in complications.

The present study analyzed published results on antibody production and antibody-related transcription factors in OM. OM alters serum Ig levels and results in the secretion of Igs into the tympanic cavity. Four transcription factors, BCL6, BLIMP-1, PAX5, and XBP1, play important roles in antibody production in the tympanic cavity.

## Figures and Tables

**Table 1:** Studies assessing the expression of antibodies in otitis media.

Authors and Reference	Study Design	Species/Type of OM	Type of Samples or Specimens	Detection Methods	Targets	Results/Conclusion
Virgil et al. [37]	Prospective study	Human (255 specimens of MEF from 165 episodes of AOM in children)	MEF vs. serum	Radial immunodiffusion	IgG, IgM, IgA	IgA: MEF > serum in almost half the patients - over 9 months of age: culture(−) was dominant in case of MEF > serum IgA IgG and IgM: MEF < serum The MEF of AOM represents primarily a secretory response to inflammation rather than a transudate.
Sloyer et al. [38]	Prospective study	Human (61 AOM childrenl)	MEF, serum	IFA, IHA	IgG, IgM, IgA (pneumococcal Ab serotype 1, 3, 6, 14, 18, 19, and 23)	Serum: IgG, IgM: predominantly detected MEF: equally detected all three classes Approximately 25% of the patients (16 of 61) had a positive immune response to their infection as evidenced by increased levels of pneumococcal antibody in the convalescent serum.
Sloyer et al. [39]	Prospective study	Human (40 AOM children)	MEF, serum	IFA, IHA	IgG, IgM, IgA (Ab to Hi)	Serum: IgG> IgM = Ig A: acute phase < convalescent phase MEF: IgG = IgA > IgM
Sloyer et al. [40]	Prospective study	Human (103 AOM childrenl)	MEF, serum	IFA, radial immunodiffusion	IgG, IgM, IgA (antibody to measles, mumps, rubella, and polio-1)	IgA: MEF > serum IgG: MEF < serum Mean specific MEF IgA titer: immunized > unimmunized
Sloyer et al. [33]	Prospective study	Human (80 patients with AOM)	MEF (cleared vs. not cleared)	Indirect fluorescent antibody, radioimmunoassay	IgG, IgM, IgA (Hi, Spn)	Abs concentration: cleared MEF > not cleared MEF Clearing of the MEF in patients with AOM due to Spn or Hi was significantly associated with the presence and concentration of specific Abs in the MEF at the time of diagnosis.
Ren et al. [41]	Prospective study	Human: 35 AOM and 149 controls	Serum	Western blot, ELISA	Serum Ab response to Mcat (OMP CD, OppA, Msp22, Hag and PilA2)	Serum IgG in all cases: Msp22 = OppA > OMP CD = Hag = PilA2. Serum antibody to Mcat increased with age in naturally immunized children age 6–30 months following Mcat NP colonization and AOM. In AOM group: IgG against OMP CD: acute phase < convalescent phase High antibody levels against OppA, Msp22, and Hag correlated with reduced carriage.
Kaur et al. [42]	Prospective cohort study	Human (137 AOM)	Serum, MEF, NW	ELISA, Western blot	IgG, IgA, sIgA	IgG: NW < MEF ≈ serum IgA: NW > MEF ≈ serum sIgA: MEF (+) IgA in MEF: originated from serum > NW
Kaur et al. [43]	Prospective study (3.5 years)	Human: 34 AOM vs. 35 rAOM vs. 25 AOMTF	Serum	ELISA	Serum IgG antibody titers of 5 different Spn proteins (PhtD, LytB, PcpA, PhtE, and Ply)	(1) Acute phase: IgG to PhtD, LytB, PhtE, Ply: rAOM < AOM = AOMTF (2) Convalescent phase: IgG to PhtD, LytB, PhtE, Ply: rAOM = AOMTF < AOM Otitis-prone and AOMTF children mount less of an IgG serum antibody response as compared with non-otitis-prone children to Spn proteins after AOM.
Kaur et al. [44]	Prospective study (3.5 years)	Human: 26 AOM vs. 32 rAOM vs. 27 AOMTF	Serum	ELISA	Serum Ab response to outer membrane protein D, P6, OMP26 of NTHi	(1) Acute phase: IgG against PD: rAOM < other two groups IgG against P6, OMP26: rAOM < AOMTF (2) Convalescent phase: rAOM and AOMTF: no change in total IgG against all the three proteins AOM: increased to PD The data on acute sera of otitis prone vs. non-otitis prone children and the acute-to-convalescence response in non-otitis prone children point to a possible link of anti-PD to protection. Further, otitis prone children should be evaluated for their responses to PD, P6, and OMP26 vaccine antigens of NTHi.
Veenhoven et al. [34]	Prospective study	Human (365 AOM children: rAOM vs. non-rAOM)	Serum	radial immunodiffusion	IgG (IgG1, IgG2), IgM, IgA	IgG (IgG1, IgG2), IgM, IgA: rAOM < non-rAOM In rAOM groups (compared with normal value): IgG, IgM, IgA, IgG1; increased levels IgG2: decreased levels Lower Ig levels in rAOM children suggest a generalized decreased Abs response in rAOM children.
Corscadden et al. [45]	Cross-sectional study	Human (166 rAOM children vs. 61 healthy controls)	Serum, MEF	multiplex bead-based assay, microsphere-based flow cytometric assay	IgG, IgG1, and IgG2 (against 11 pneumococcal polysaccharides: 1, 4, 5, 6B, 7F, 9V, 14, 18C, 19A, 19F, and 23F 1)	IgG and IgG1 against serotype 5: rAOM > control All pneumococcal serotype specific IgG in rAOM: serum = MEF
Wiertsema et al. [46]	Cross-sectional study	Human (172 rAOM vs. 63 controls)	Serum	Multiplex bead assay	IgG: against 4 pneumococcal (PspA1, PspA 2, CbpA, and Ply) and 3 NTHi (P4, P6, and PD)	IgG against NTHi P4, P6, PD: rAOM > control IgG against pneumococcal protein antigens: rAOM ≈ control
Krakau et al. [47]	Prospective study	Human: 28 adults with low IgG2 level during childhood (15: a history of rAOM during childhood vs. 13 controls)	Serum	Nephelometry, ELISA	total IgG and IgG subclasses 1–4	All Igs: rAOM = control Study subjects who had rAOM combined with low IgG2 levels during childhood had a normalized immunoglobulin pattern as adults.
Freijd et al. [48]	Prospective study	Human (15 pOME children vs. 15 age matched healthy control children vs. 15 healthy adults)	Plasma samples	ELISA	Spn Abs of different IgG subclasses	Adults: IgG1 < IgG2 Children: IgG1 > IgG2 (pOME < control)
Verhaegh et al. [49]	Prospective cohort study	Human (rAOM vs. cOME)	Serum, MEF	Luminex xMAP technology	IgG, IgM, IgA (against Spn and Mcat)	Serum and MEF antigen-specific IgG, IgM, IgA: rAOM ≈ cOME Serum or MEF IgG, IgM, IgA level were not different and not related to bacterial identification in both groups Serum and MEF show strong correlation for only IgG in both groups
Shin et al. [50]	Prospective study	Human (29 p-OME vs. 32 OME children)	MEF	ELISA	IgG, IgM, IgA	IgA: pOME < OME. Lower concentrations of IgA in middle ear fluid of patients with OME may be related to OME recurrence and chronicity.
Yamanaka et al. [51]	Prospective study	Human (320 OME ears: acute, 10.3%; subacute, 16.6%; chronic, 73.1%)	MEF	ELISA	IgG, IgM, IgA	IgG-ICs: highest positive rate was found in acute cases IgA-ICs: highest positive rate was found in subacute cases neutrophil dominant type in chronic cases: highest IgG-ICs level ICs formed in the MEF might play an important role in the prolonged inflammatory process of OME through the complement activation following chemotaxis of neutrophils.
Yeo et al. [52]	Prospective study	Human (58 cOME vs. 64 controls)	MEF, serum	ELISA, nephelometry	IgG, IgM, IgA	Serum IgG, Ig M, IgA: cOME < control MEF Ig concentration–the presence of bacteria: no correlation MEF Ig concentration–serum Ig concentration: no correlation Serum Ig concentration: bacteria (+) > bacteria (−) The presence of effusion bacteria in OME may be related to systemic immunity, but the concentration of Ig in effusion fluid may not be affected by the presence of effusion bacteria.
Drake-Lee et al. [53]	Two age-matched cohorts study	Human (50 OME vs. 50 age-matched controls)	Serum	ELISA, radial immunodiffusion	Total IgG, IgG subclass, total IgA, IgA subclass	Total IgA, IgA2, total IgG, IgG2: OME ≈ control normal antibody response between both groups of patients.
Chung et al. [54]	Prospective study	Human (27 mucoid OME vs. 18 serous OME)	MEF	Immunoblot assay	sIgA	sIgA: mucoid OME > serous OME
Takada et al. [55]	Prospective study	Human (59 OME children)	MEF, serum	ELISA	IgG, IgM, IgA, and sIgA antibodies specific to outer membrane antigens of Mcat	Serum: IgG > IgA > IgM MEF: IgG > IgM > sIgA > IgA All Ig: MEF > serum IgG and IgM in MEF: acute phase > subacute/chronic phase sIgA in MEF: acute phase < subacute/chronic phase IgG in serum or MEF: recurrent/persistent OME group < nonrecurrent/non-persistent OME group Decreased serum and MEF IgG antibody levels specific to outer membrane antigens of Mcat may lead to failure to eliminate this organism, resulting in persistent and/or recurrent appearance of MEF.
Faden et al. [56]	Prospective study	Human (14 OME)	Serum, MEF	Ab assay by 96-well microtiter plate	IgG, IgM, IgA	Serum: IgG > IgM > IgA MEF: IgG > IgA > IgM The IgG- and IgA-specific antibody present in middle ear effusions appeared to represent local production rather than passive diffusion from the systemic circulation.
Lasisi et al. [57]	Prospective study	Human (20 cSOM vs. 17 aSOM vs. 15 controls)	MEF, serum	ELISA	IgG, IgM	Serum IgG: cSOM > control > aSOM MEF IgG: cSOM > aSOM Serum IgM: aSOM > cSOM> control MEF IgM: aSOM > cSOM
Lasisi et al. [58]	Prospective study	Human: 20cSOM vs. 17 aSOM vs. 15 controls	Serum, MEF	Radial immunodiffusion	IgE	Serum IgE: cSOM > aSOM > control MEF IgE: cSOM > aSOM MEF/serum IgE ratio: cSOM > aSOM Serum and MEF showed correlation in cSOM. Allergy appears to play a contributory role in CSOM and elevated IgE in the MES suggests a likely mucosal response.

OM, otitis media; MEF, middle ear fluid; Ig, immunoglobulin; AOM, acute otitis media; p-OME, prone-otitis media with effusion; OME, otitis media with effusion; ELISA, enzyme-linked immunosorbent assay; ICs: immune complexes; Hi, *Haemophilus influyenzae*; Spn, *Streptococcus pneumoniae*; Abs, antibodies; Mcat, *Moraxella catarrhalis*; sIgA, secretory IgA; rAOM, recurrent acute otitis media; cOME, chronic otitis media with effusion; cSOM, chronic suppurative otitis media; aSOM, acute suppurative otitis media; IFA, indirect fluorescent antibody test; IHA, indirect hemagglutination; rAOM, recurrent acute otitis media; PspA, *Streptococcus pneumoniae* antigens pneumococcal surface protein A family; CbpA, choline binding protein A; Ply: pneumolysin; NTHi, nontypeable *Haemophilus influenza*; P4, Protein 4; P6, Protein6; PD, Protein D; AOMTF, acute otitis media treatment failure; OMP, outer membrane protein; OppA, oligopeptide permease A; Hag, hemagglutinin; Msp, Moraxella surface protein; PilA2, PilA clade 2; Pht, pneumococcal histidine triad proteins; LytB, a murein hydrolases; PcP, a choline binding protein; NW, nasal wash.
